# Music With and Without Lyrics Increases Motivation, Affective Valence, and Arousal During Moderate-Intensity Cycling

**DOI:** 10.3390/bs16081357

**Published:** 2026-08-07

**Authors:** Ryan L. Olson, Daniel N. Marshall, Melissa A. Materia

**Affiliations:** 1 Department of Kinesiology, Health Promotion and Recreation, University of North Texas, Denton, TX 76203, USA; 2 Department of Psychology, University of North Texas, Denton, TX 76203, USA

**Keywords:** music, lyrics, music with lyrics, motivation, affective valence, arousal, perceived exertion, exercise

## Abstract

Music is used during exercise to enhance psychological and physiological responses, including increased motivation, improved affective valence, optimized arousal, and reduced perceived exertion. Although researchers have identified several musical elements that may moderate these effects, the contribution of lyrics remains poorly understood. The present study examined the effects of lyrics on motivation, affective valence, arousal, and perceived exertion during moderate-intensity cycling. Thirty college students (*M*_age_ = 21.0 ± 2.9 years) completed 8-min bouts of moderate-intensity cycling under three counterbalanced conditions: music with lyrics (ML), music without lyrics (MNL; an instrumental version of the same track), and a no-music metronome control (MC). Motivation, affective valence, arousal, and perceived exertion were sampled at the end of a 6-min warm-up and at four in-task time points (8, 10, 12, and 14 min). Both music conditions produced significantly higher motivation, affective valence, and arousal than the metronome control at the in-task time points (8–14 min), with no differences between ML and MNL at any time point. Significant Condition × Time interactions indicate that these condition differences did not apply uniformly across the analytic window. Perceived exertion increased over time but did not differ across conditions. Under these specific conditions (a single, highly familiar popular track, an 8-min moderate-intensity cycling bout, and a metronome overlaid across all conditions), no additional psychological benefit of lyrical content was detected beyond that provided by the music itself. These results should not be generalized as evidence that lyrics have no effect on exercise-related psychological responses.

## 1. Introduction

Music is frequently used during exercise to enhance psychological and physiological responses (Karageorghis & Priest, 2012a, 2012b; Sloboda et al., 2009). Research demonstrates that music enhances motivation (Hutchinson et al., 2011), affect (Elliott et al., 2004; Suwabe et al., 2021), arousal (Frith & Loprinzi, 2018; Ghaderi et al., 2009), and may reduce perceived exertion (Clark et al., 2021; Potteiger et al., 2000). Although several elements of music (e.g., tempo, rhythm, sound, loudness, harmony) have been investigated (Karageorghis et al., 2008; Yamamoto et al., 2003), the specific contribution of lyrics remains unclear.

In the model proposed by Karageorghis (2016), lyrics are considered an intrinsic musical factor that interacts with personal (e.g., musical preference, song familiarity) and situational (e.g., exercise mode and intensity) variables. Qualitative and descriptive work suggests lyrics can enhance motivation (Karageorghis et al., 2013), affective valence (Priest & Karageorghis, 2008), and arousal (Urakawa & Yokoyama, 2005), and reduce perceived exertion (Gfeller, 1988), with effects shaped by exercise context, lyrical interpretation, and empathy or imagery processes (Bishop et al., 2007).

Beyond a general reference to semantic content, lyrics are theorized to influence exercise-related psychological responses through at least three interrelated mechanisms. First, lyrics may modulate attentional focus via dissociation by drawing attention externally toward the song’s words and, by extension, away from interoceptive cues of effort and fatigue (Hutchinson & Karageorghis, 2013; Rejeski, 1985). Second, lyrics carry explicit emotional and motivational content including themes of effort, energy, perseverance, or empowerment that may prime goal-directed cognition and reinforce affective states congruent with continued exercise (Karageorghis, 2016; Priest & Karageorghis, 2008). Third, familiar lyrics can evoke autobiographical or imagery-based associations that intensify emotional engagement independent of any explicit motivational message (Bishop et al., 2007; Ali & Peynircioğlu, 2006). Each mechanism predicts that lyric-containing music should produce psychological responses equal to or greater than the same music without lyrics. Identifying which, if any, are operative during acute exercise may require designs that hold acoustic content constant across lyric and instrumental versions of the same track.

Unlike structural musical features (e.g., tempo, rhythm), lyrics are not standardized, and their motivational impact depends on semantic content, language familiarity, and personal interpretation (Ali & Peynircioğlu, 2006; Juslin, 2009). This heterogeneity likely contributes to the small literature on lyrics in exercise. In one of the earliest empirical comparisons, Sanchez et al. (2014) examined music with lyrics, without lyrics, and no music during 6 min of cycling at 75% of maximum heart rate. Cadence increased in both music conditions relative to control, with small late-bout increases in the lyric condition. Perceived exertion did not differ across conditions, which is consistent with the dissociative properties of music (Hutchinson et al., 2011; Rejeski, 1985). Contrary to broader meta-analytic findings (Boutcher & Trenske, 1990; Terry et al., 2020), Sanchez and colleagues found no affective valence differences across groups, suggesting that single-track comparisons at a fixed intensity may be underpowered to detect affective effects (see also Marshall, 2017).

Baranie et al. (2015) measured perceived exertion in 23 non-athletic adults following 5 min of vigorous treadmill running (~80% age-predicted HR_max_) under music-with-lyrics, music-without-lyrics, and no-music conditions. Both music conditions reduced perceived exertion relative to the control, with no difference between the lyric and non-lyric conditions. Perceived exertion was sampled only once (post-exercise, after 20–30 min of warm-up); however, and no psychological outcomes beyond rate of perceived exertion (RPE) were assessed, precluding inferences about baseline state or in-task change.

These inconsistent findings likely reflect methodological limitations (Marshall, 2017). Sanchez et al. (2014) standardized resistance but allowed self-selected cadence, which may have masked condition-related affective changes. Furthermore, uncontrolled intensity drift above ventilatory threshold may further attenuate affective response (Ekkekakis et al., 2011). Both Sanchez et al. (2014) and Baranie et al. (2015) also sampled affective or perceptual outcomes only before and after exercise, preventing researchers from drawing conclusions about in-task changes. Additionally, both rated lyric motivational content at rest rather than during exercise at a comparable intensity, which may limit validity. Finally, to our knowledge, few studies have directly examined the effects of lyrics on motivation during exercise, despite the centrality of motivation to the music-performance relationship.

The primary aim of the present study was to examine the effects of lyrics on motivation during moderate-intensity cycling, with state motivation defined as participants’ moment-to-moment willingness to continue the task (Tenenbaum et al., 2007). We hypothesized that both music conditions would increase motivation relative to control, and that the lyric condition would produce a larger effect than the instrumental condition because achievement-oriented lyrical content (e.g., effort, energy, perseverance) provides an additional semantic cue supporting goal-directed cognition during exercise (Karageorghis, 2016; Priest & Karageorghis, 2008). A secondary aim was to examine effects on affective valence, arousal, and perceived exertion. We expected both music conditions to elevate affective valence and arousal relative to the control, with larger effects in the lyric condition. We also expected no condition differences in perceived exertion given the standardized workload and brief (8-min) duration.

## 2. Materials and Methods

### 2.1. Part 1: Music Selection

A single track was selected for use in the Part 2 experimental trials (procedures adapted from Marshall, 2017). Researchers first identified five candidate tracks from the list of songs recommended to accompany cycling tasks in Karageorghis (2016). These five songs were the highest-rated tracks for motivational lyric content among the songs suitable for the target cycling cadence. The full list of candidate songs and BMRI-2 scores can be found in Table 1. To rate these tracks, twenty-eight students (*M*_age_ = 22.11, *SD* = 1.89 years) volunteered for Part 1. Part 1 participants met the same inclusion/exclusion criteria as Part 2 (see Section 2.2.2) and were fully independent of the Part 2 sample. The release dates of the five candidate tracks varied from 1978–2015. All Part 1 and Part 2 participants were familiar with four of the five tracks (released 2009–2015), and none were familiar with *Instant Replay* by Dan Hartman (1978). In Part 1, participants completed one continuous cycling bout in which the five candidate tracks were presented in a randomized order for 2 min each (approximately 10 min of cycling in total, plus a brief warm-up used to bring the participant to the target exertion). Exercise intensity was based on Borg’s Ratings of Perceived Exertion (RPE) Scale (Borg, 1982). Depending on the track being played, participants maintained a steady cadence between 60–65 revolutions per minute (RPM; set to half the BPM of the track; see Table 1) while incremental adjustments of resistance were made on the ergometer. Only after each participant reached a target RPE of 12–13 (Garber et al., 2011) did they begin listening to the sequence of five tracks. After listening to each track for 2 min, and while remaining seated and pedaling on the ergometer, participants rated the motivational quality of the track using the Brunel Music Rating Inventory-2 (BMRI-2; Karageorghis et al., 2006). The BMRI-2 is a 6-item instrument presented on a 7-point Likert scale, anchored by 1 (strongly disagree) and 7 (strongly agree), designed to measure the motivational qualities of music for use in exercise. Participants provided verbal responses to each item, which were then confirmed by experimenters prior to recording.

In addition to the original six items on the BMRI-2, two items assessing vocals and lyrics were included to ensure that the lyrical content of the tracks was perceived as motivational. As with all questions in the BMRI-2, participants were asked their level of agreement with a statement based on attributes of the music. Added item 1 stated, “the vocals (i.e., singing) of this music would motivate me during exercise” while added item 2 stated, “the lyrics (i.e., words accompanying the music) would motivate me during exercise.” In the present study, participants were informed that the term “motivate” meant it would “make you want to exercise harder and/or longer during a cycling task.” Finally, the total sum BMRI-2 score (range: 6–42) and the sum of the two added lyric/vocal items (range: 2–14) were calculated for each track. The track with the highest total BMRI-2 score (*M* = 33.31, *SD* = 8.88) and highest lyric score (*M* = 10.68, *SD* = 3.20) was selected for the experimental trials (*Boom Boom Pow* by the Black Eyed Peas).

### 2.2. Part 2: Experimental Trials

#### 2.2.1. Power Analysis

An a priori power analysis was conducted using G*Power 3.1.9.2 (Faul et al., 2009) for the primary contrast, a 3 (Condition: ML, MNL, MC) × 5 (Time) repeated-measures ANOVA on state motivation. The expected effect size was anchored to a meta-analysis of music in exercise and sport (Terry et al., 2020), which reported a pooled standardized mean difference of *d* = 0.48 for music-versus-control comparisons on psychological outcomes. Converting this estimate to Cohen’s *f* for a three-condition omnibus contrast yields *f* = 0.24. With α = 0.05, 1 − β = 0.80, an assumed within-subject correlation of *r* = 0.50, and a Greenhouse-Geisser nonsphericity correction of ε = 0.80, G*Power indicated that a minimum of 27 participants was required to detect the predicted effect on the omnibus Condition × Time interaction. The number of participants was increased to 30 to balance counterbalancing of condition order across participants. A post hoc sensitivity analysis with the achieved sample (*n* = 30) confirmed that the present design has 80% power to detect effects as small as *f* = 0.18 for the Condition × Time interaction under the same assumptions, indicating adequate sensitivity for the omnibus contrast. Importantly, this a priori estimate was anchored to the well-established music-versus-control effect (*d* = 0.48; Terry et al., 2020) and was not derived from a lyric-specific ML-versus-MNL contrast. Because any lyric-specific effect is theoretically expected to be smaller than the omnibus music-versus-control effect on which the power analysis was based, the present study was not specifically powered to detect small ML-versus-MNL differences. Non-significant ML-MNL contrasts should not be interpreted as evidence of equivalence or as evidence for the absence of a lyric-specific effect. They indicate only that no reliable ML-MNL difference was detected under this design and sample.

#### 2.2.2. Participants

Thirty college students (19 females; 11 males) aged between 18–35 years old (*M*_age_ = 21.03, *SD* = 2.94 years) were recruited from the local university via recruitment emails and flyers (sample as originally reported by Marshall, 2017). Both recruitment emails and flyers provided information on the study requirements, including inclusion criteria, the completion of questionnaires, total time commitment and number of sessions, lab location, and lab contact information. Full participant demographic information can be found in Table 2. Inclusion criteria included: (1) men and women aged 18–35 years; (2) no physical limitations or contraindications to exercise; and (3) reported regularly listening to music during exercise. Participants were excluded if they met one or more of the following criteria: (1) current or present history of cardiovascular disease; (2) past or present history of psychiatric or neurological disorder; (3) currently taking medications that would prevent them from completing moderate-to-vigorous intensity exercise; and/or (4) pregnancy or considering becoming pregnant in women. Prior to each session, enrolled participants were screened for regular sleep patterns, stimulant use (e.g., caffeine and tobacco), meal consumption, exercise participation, stress levels, and current mood. Any subjects who provided irregular responses relative to their normal daily activities were rescheduled. The university’s accredited Institutional Review Board approved all research procedures, and all subjects provided written informed consent prior to study participation. Additionally, all research was performed in accordance with local and federal regulations and was completed in accordance with the Declaration of Helsinki.

#### 2.2.3. Heart Rate (HR) and Intensity

HR_max_ was estimated using the Fox equation (220 − age) as recommended by ACSM guidelines for establishing exercise intensity zones in the general population (Garber et al., 2011). We acknowledge that population-specific regression equations may provide superior estimates in certain samples (Garber et al., 2011). However, Shookster et al. (2020) directly compared several age-predicted HR_max_ equations against measured maximal HR values and identified the Fox equation as the most accurate readily available option in the general population when a graded exercise test is not feasible. Because a graded exercise test was outside the scope of the present study protocol, the Fox equation was used to derive individualized target heart rate ranges.

#### 2.2.4. Rate of Perceived Exertion (RPE)

The in-task perception of physical exertion was measured using Borg’s 15-point scale (Borg, 1982), which ranges from 6 to 20 with verbal anchors at 7 (very, very light), 9 (very light), 11 (fairly light), 13 (somewhat hard), 15 (hard), 17 (very hard), and 19 (very, very hard). Participants were given the same verbal instructions as recommended by Borg (1998) prior to testing during both Parts 1 and 2 (measures as previously described by Marshall, 2017). The scale demonstrates strong validity and reliability in exercise settings (Borg, 1998; Chen et al., 2002).

#### 2.2.5. Motivation

An 11-point Motivation Scale (MS; Tenenbaum et al., 2007) assessing the direction and intensity of effort was used to measure state motivation. Participants were asked, “How motivated are you to keep going?” at the end of each 2-min block. The scale is anchored at 0 (not at all motivated), 5 (moderately motivated), and 10 (extremely motivated). It demonstrates construct validity as a single-item measure of motivation (Tenenbaum et al., 2007).

#### 2.2.6. Affective Valence

In-task affective valence was assessed using the 11-point Feeling Scale (FS; Hardy & Rejeski, 1989). The FS is a single-item, bipolar scale of pleasure and displeasure with anchors at −5 (very bad), −3 (bad), −1 (fairly bad), 0 (neutral), +1 (fairly good), +3 (good), and +5 (very good). The FS is widely used and validated for assessing affective valence during exercise (Ekkekakis, 2003; Hardy & Rejeski, 1989).

#### 2.2.7. Arousal

The 6-point Felt Arousal Scale (FAS; Svebak & Murgatroyd, 1985) was used to measure in-task arousal levels. The FAS is a single-item scale with anchors at 1 (low arousal) and 6 (high arousal). The FAS has demonstrated acceptable validity in exercise research (Astorino et al., 2012; Ekkekakis et al., 2008).

#### 2.2.8. Procedures

Participants visited the laboratory on four separate occasions at approximately the same time of day, separated by at least 48 h (procedures adapted from Marshall, 2017). During the familiarization session (day 1), participants were screened for eligibility and provided written informed consent. Participants then completed health history and physical activity readiness (PAR-Q) questionnaires to collect demographic data and ensure exercise safety, respectively. Participants were then escorted to the cycle ergometer (Monark Ergomedic 828E, Varberg, Sweden) where adjustments were made for optimal comfort (e.g., seat height and pedal settings). Values were recorded and used for the remaining sessions. Participants were also introduced to the psychological assessments of motivation (MS), affective valence (FS), arousal (FAS), and perceived exertion (RPE). Prior to leaving the lab, participants were instructed to wear athletic attire that would provide them comfort and free range of motion to exercise for up to 20 min during their remaining sessions.

**Figure 1:**
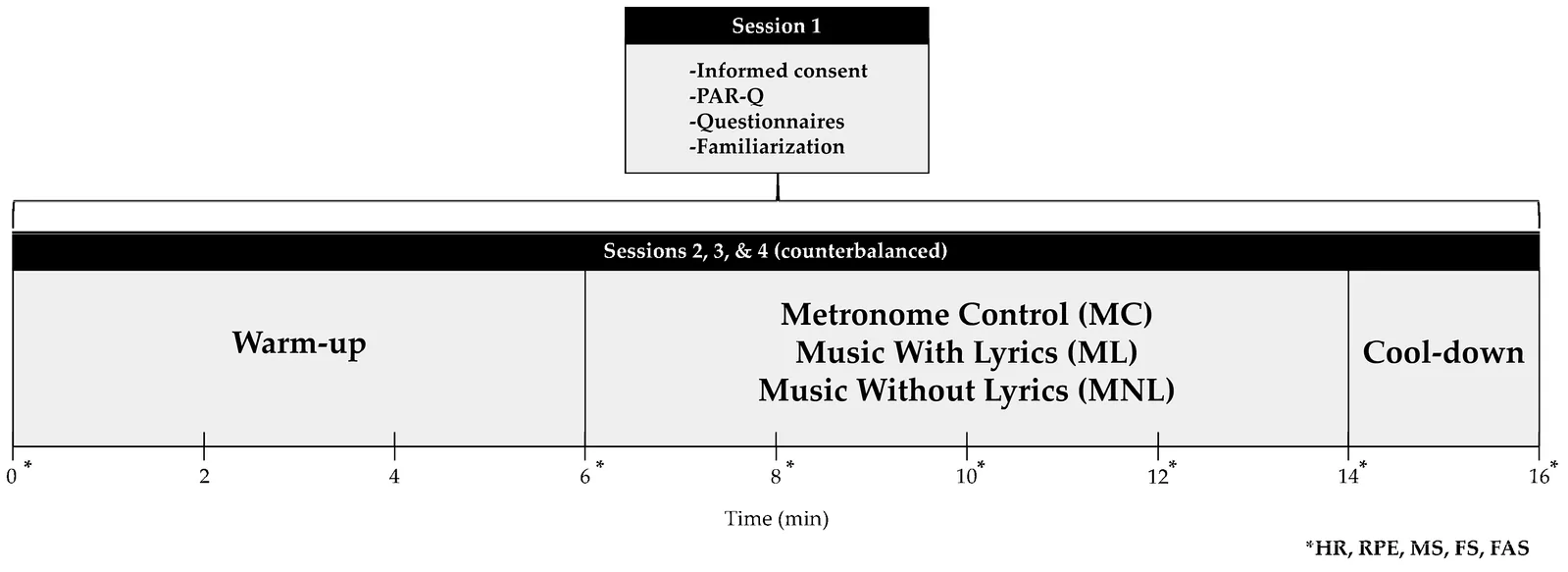
Experimental study design.

During days 2–4, a within-subjects experimental design was utilized to examine the effects of music with and without lyrics on the primary outcome, motivation. Secondary outcomes included affective valence, arousal, and perceived exertion responses. Participants completed music with lyrics (ML), music without lyrics (MNL), and no music control (MC) conditions on three separate days in counterbalanced order. The 8-min exercise bout length was selected to balance (a) providing sufficient in-task sampling of psychological states (five measurement points at 2-min intervals), (b) minimizing fatigue and drift at moderate intensity, and (c) avoiding boredom from looping the same track multiple times. Furthermore, this timing is consistent with durations used in prior music-exercise studies (e.g., Karageorghis et al., 2009; Sanchez et al., 2014). All three conditions were 16 min in duration and consisted of a 6-min warm-up, 8 min of continuous exercise at moderate-intensity, and a 2-min cool-down. All outcome measures were assessed throughout each condition at seven time points (0, 6, 8, 10, 12, 14, and 16 min; Figure 1). Statistical analyses were restricted to the five time points within the experimental manipulation window, one pre-condition baseline taken at the end of the warm-up (6 min) and four in-task assessments (8, 10, 12, and 14 min). This approach was adopted because participants were neither exposed to the auditory condition nor cycling at the target intensity at the pre-warm-up (0 min) or post-cool-down (16 min) time points. Including those measurements could inflate outcomes without informing whether the experimental conditions produced differential effects during the exercise bout.

The testing area was a 20′ × 20′ temperature-controlled room with access to a cooling fan for comfort, water for hydration, and a towel. At the start of each session, participants were again briefed on each psychological assessment and instructed to respond verbally throughout the test session. Responses were confirmed by the experimenter prior to recording. Large printed versions of each scale were mounted on the wall approximately 3′–5′ from the ergometer based on participant viewing preference. A metronome was played at 65 BPM throughout the entire session (warm-up, 8-min experimental bout, and cool-down). Participants were explicitly instructed to synchronize their pedaling with the metronome at all times, and the metronome tempo was aligned to half the tempo of the selected track (130 BPM ÷ 2 = 65 BPM). Because participants were actively matching their cadence to the metronome and the metronome was aligned with the music tempo, this study is best characterized as a synchronous music paradigm (Karageorghis et al., 2011). During the 6-min warm-up, experimenters began at a low resistance and incrementally increased resistance every 30–60 s based on real-time heart rate telemetry until the participant reached the lower bound of their target range (64% HR_max_). This process was standardized across participants by using the same increment schedule and target range. In all cases, the 8-min experimental bout began when the 6-min warm-up period elapsed. Most participants reached their target heart rate range earlier than 6 min and continued cycling within the target range until the warm-up period ended. No participant required more than 6 min to reach the target range. Using a fixed 6-min warm-up ensured consistent pre-condition timing across participants regardless of individual differences in fitness, while also minimizing acute cardiovascular transients at exercise onset. During the 8-min experimental bout, participants heard the selected song in its original form (ML condition), an instrumental version of the same song (MNL condition), or the metronome alone (MC condition). The music was overlaid on the metronome during ML and MNL, while only the metronome played during MC. The metronome was retained across all three conditions to keep cadence and auditory background stimulation as constant as possible across ML, MNL, and MC, so that condition differences could be attributed to lyrical content rather than to differences in cadence variability or the presence of a non-music auditory cue. This design choice also equated auditory stimulation such that condition differences could not be attributed to the presence versus absence of the metronome itself. Whether the constant metronome overlay independently influenced attentional allocation or psychological state cannot be evaluated in the present design because it was applied identically in all three conditions; however, any such effect should be equivalent across ML, MNL, and MC and therefore should not have biased the between-condition comparisons of primary interest. We acknowledge that overlaying a metronome on the music tracks departs from typical listening contexts and may reduce ecological validity. Following the 8-min bout, the music faded out and participants continued pedaling in synchrony with the metronome during the 2-min cool-down. During the cool-down, the experimenter progressively reduced resistance while participants maintained the metronome-paced cadence, thus mechanical workload, along with heart rate and exertion levels, declined toward baseline by the end of the 2-min cool-down period. Throughout the 8-min experimental bout itself, the experimenter continued to make small adjustments to the resistance as needed to keep participants within their individualized target heart rate range.

The lyric (ML) and instrumental (MNL) versions of the selected track were commercially released, professionally produced versions of the same recording. No post hoc vocal removal or software-based vocal isolation was applied, and no backing-vocal traces were present in the MNL version above what appears in the released instrumental mix. The two versions were tempo-identical by virtue of being the same underlying recording. Prior to use, both files were objectively matched for loudness by normalizing to a common integrated loudness target (measured in loudness units full scale; LUFS) using digital audio workstation software. Playback volume was then set to a single verified level on the speaker system (Logitech Inc., San Jose, CA, USA) positioned behind the ergometer, and this level was used across all sessions and conditions. Post-condition ratings of song enjoyment, familiarity, or perceived motivational quality of the ML and MNL song versions were not collected in the present study. Due to the length of the selected song (4 min), each version was played twice in succession to fill the 8-min bout. We acknowledge that replaying the same track (rather than using a second, matched track) may have attenuated any lyric-specific novelty across the second half of the bout. This approach was chosen to keep the acoustic content identical across ML and MNL, thereby isolating the presence/absence of lyrics. Motivation (MS), affective valence (FS), arousal (FAS), perceived exertion (RPE), and heart rate (HR) were measured at the beginning and end of the warm-up, at each 2-min interval during exercise, and at the end of the cool-down. Following completion of the final test session (day 4), participants were debriefed on the study.

#### 2.2.9. Data Analysis

Descriptive statistics were performed on participant demographic, cycling history, and music preference data using SPSS version 29 (IBM Corp., Armonk, NY, USA). Each outcome variable of interest (MS, FS, FAS, RPE, and HR) was submitted to a 3 (Condition: ML, MNL, MC) × 5 (Time: 6, 8, 10, 12, 14 min) repeated-measures analysis of variance (RM-ANOVA) with a family-wise α level of 0.05. Given the within-subjects factorial design, the sensitivity of the univariate RM-ANOVA to violations of sphericity, and the small sample size relative to the number of repeated measures, we adopted the multivariate approach to repeated measures (Wilks’ Λ) as our primary omnibus test. All *F* statistics and degrees of freedom reflect the multivariate output (e.g., for the Condition main effect with *k* = 3 levels and *n* = 30, *F*(*k* − 1, *n* − *k* + 1) = *F*(2, 28); for the Time main effect with *t* = 5 levels, *F*(*t* − 1, *n* − *t* + 1) = *F*(4, 26); and for the Condition × Time interaction, *F*((*k* − 1)(*t* − 1), *n* − (*k* − 1)(*t* − 1)) = *F*(8, 22)). The multivariate approach does not assume sphericity, so Mauchly’s test and Greenhouse-Geisser or Huynh-Feldt corrections were not required. Significant omnibus effects were decomposed with pairwise Bonferroni-corrected contrasts within each outcome. Corrections were applied within each outcome rather than across all five outcomes because the five dependent variables (MS, FS, FAS, RPE, HR) index conceptually distinct constructs and were pre-specified as separate hypotheses, and because HR functioned as a manipulation check rather than a primary outcome. Applying a family-wise correction across five outcomes would have imposed an unusually stringent α level (approximately 0.01 per family) that is not conventional in this literature and would have obscured effects on the theoretically central primary outcomes. We acknowledge, however, that not correcting across outcomes leaves the possibility that marginal findings could reflect an inflated Type I error rate. Results should therefore be interpreted with caution and treated as tentative until replicated. When a significant Condition × Time interaction was obtained, simple effects of condition were evaluated at each time point. Marginal main effects are also reported below to characterize the overall condition and time patterns, but interpretation is guided primarily by the interaction. To directly address the theoretically central ML–MNL contrast, planned paired comparisons of ML and MNL, averaged across the 6–14 min analytic window, are additionally reported for each outcome with 95% confidence intervals and standardized effect sizes (Cohen’s *d_z_* for paired data). Effect sizes for the omnibus RM-ANOVA are reported as partial eta squared (*η*^2^*_p_*).

## 3. Results

Preliminary analyses of participants from the experimental trials revealed no significant sex differences in BMI, age, prior music listening frequency, song enjoyment, or cycling history. Similarly, there were no significant sex differences in MS, FS, FAS, RPE, or HR responses. Thus, subsequent analyses were collapsed across sex.

### 3.1. Measures

#### 3.1.1. HR (Intensity Manipulation Check)

**Figure 2:**
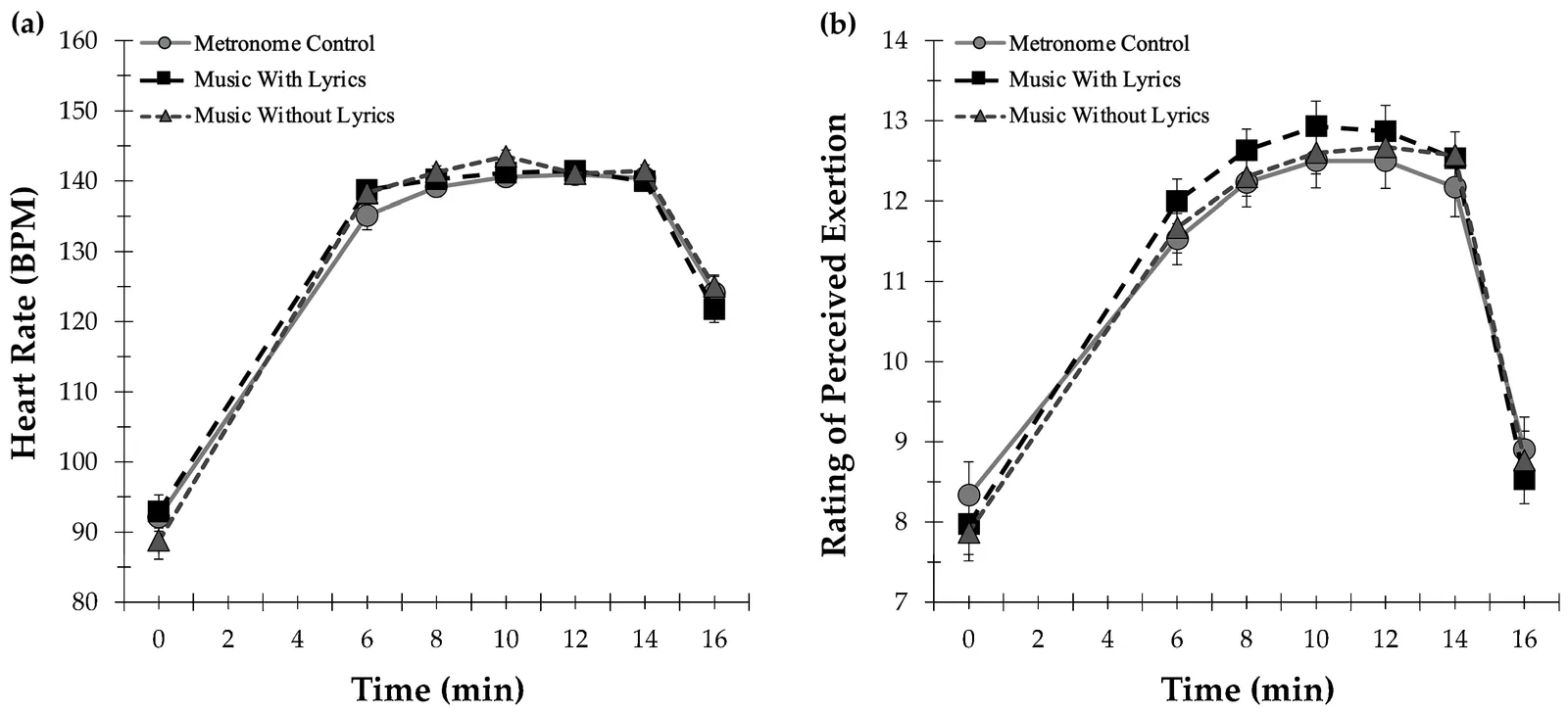
Average (**a**) heart rate (BPM) and (**b**) perceived exertion (RPE) across a 16-min cycling trial performed during music with lyrics, music without lyrics, and metronome control conditions. For clarity of interpretation, the y-axes are truncated and do not reflect anchor points.

Heart rate was retained as a manipulation check rather than a primary dependent variable, as it was used to standardize exercise intensity across conditions. As expected, average HR during exercise fell within the calculated 64–76% HR_max_ range (*M* = 140.2, *SE* = 3.27 BPM) for all conditions. The RM-ANOVA did not reveal a Condition main effect for HR, *F*(2, 28) = 2.07, *p* = 0.15, *η*^2^*_p_* = 0.13, suggesting that HR was similar between all three experimental conditions. A Time main effect, *F*(4, 26) = 7.41, *p* < 0.001, *η*^2^*_p_* = 0.53, showed an increase in HR from the end of the warm-up period (6-min time point) compared to a relatively stable HR throughout exercise (8-, 10-, 12-, 14-min time points). No Condition × Time interaction was found, *F*(8, 22) = 0.98, *p* = 0.48, *η*^2^*_p_* = 0.26 (Figure 2).

#### 3.1.2. Perceived Exertion (RPE)

A significant Time main effect, *F*(4, 26) = 8.89, *p* < 0.001, *η*^2^*_p_* = 0.58, was found for perceived exertion such that lower perceived exertion was reported at the 6-min (*M* = 11.73, *SE* = 0.24) time point compared to higher perceived exertion at the 8-min (*M* = 12.39, *SE* = 0.23), 10-min (*M* = 12.68, *SE* = 0.27), 12-min (*M* = 12.68, *SE* = 0.26), and 14-min (*M* = 12.42, *SE* = 0.28) time points. No significant Condition main effect, *F*(2, 28) = 1.59, *p* = 0.22, *η*^2^*_p_* = 0.10, or Condition × Time interaction was observed, *F*(8, 22) = 0.26, *p* = 0.97, *η*^2^*_p_* = 0.09 (Figure 2).

#### 3.1.3. Motivation (MS)

**Figure 3:**
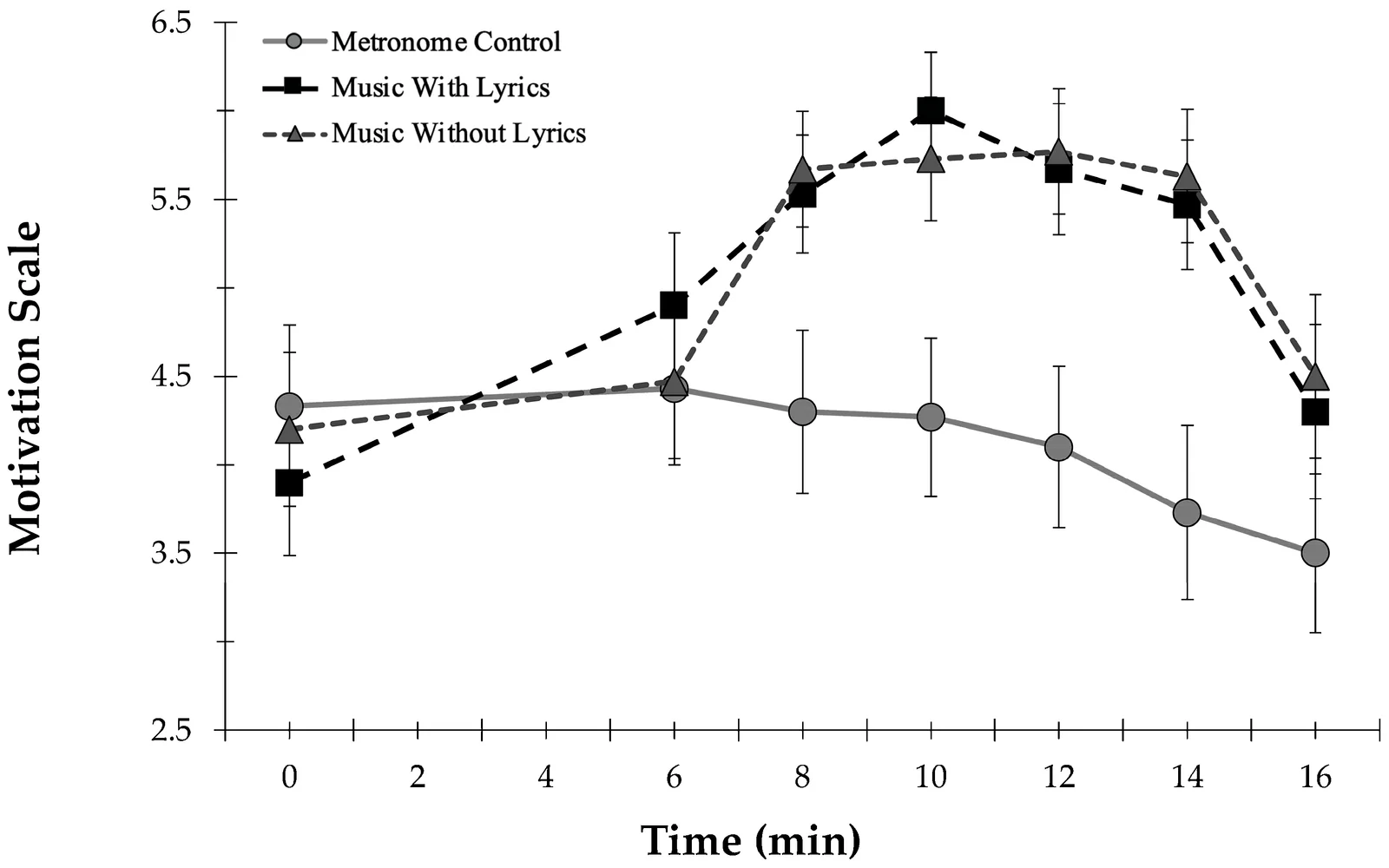
Average motivation (MS) across a 16-min cycling trial performed during music with lyrics, music without lyrics, and metronome control conditions. For clarity of interpretation, the *y*-axis is truncated and does not reflect anchor points.

The RM-ANOVA for the MS revealed a significant Condition main effect, *F*(2, 28) = 12.96, *p* < 0.001, *η*^2^*_p_* = 0.48, such that motivation was reported highest in the ML (*M* = 5.51, *SE* = 0.34) and MNL (*M* = 5.45, *SE* = 0.35) conditions relative to the MC (*M* = 4.17, *SE* = 0.44) condition. There was no difference found between the two music conditions. Additionally, a significant Time main effect, *F*(4, 26) = 10.11, *p* < 0.001, *η*^2^*_p_* = 0.61, showed that participants felt significantly less motivated at 6-min (*M* = 4.60, *SE* = 0.40) and 14-min (*M* = 4.94, *SE* = 0.37) time points compared to 8-min (*M* = 5.17, *SE* = 0.34), 10-min (*M* = 5.33, *SE* = 0.33), and 12-min (*M* = 5.18, *SE* = 0.35) time points. These main effects were superseded by a significant Condition × Time interaction, *F*(8, 22) = 4.85, *p* = 0.002, *η*^2^*_p_* = 0.64. Decomposition of this interaction revealed motivation was significantly higher in ML and MNL conditions at the 8-, 10-, 12-, and 14-min time points relative to the MC condition. There were no differences between conditions found at the 6-min time point (Figure 3).

#### 3.1.4. Affective Valence (FS)

**Figure 4:**
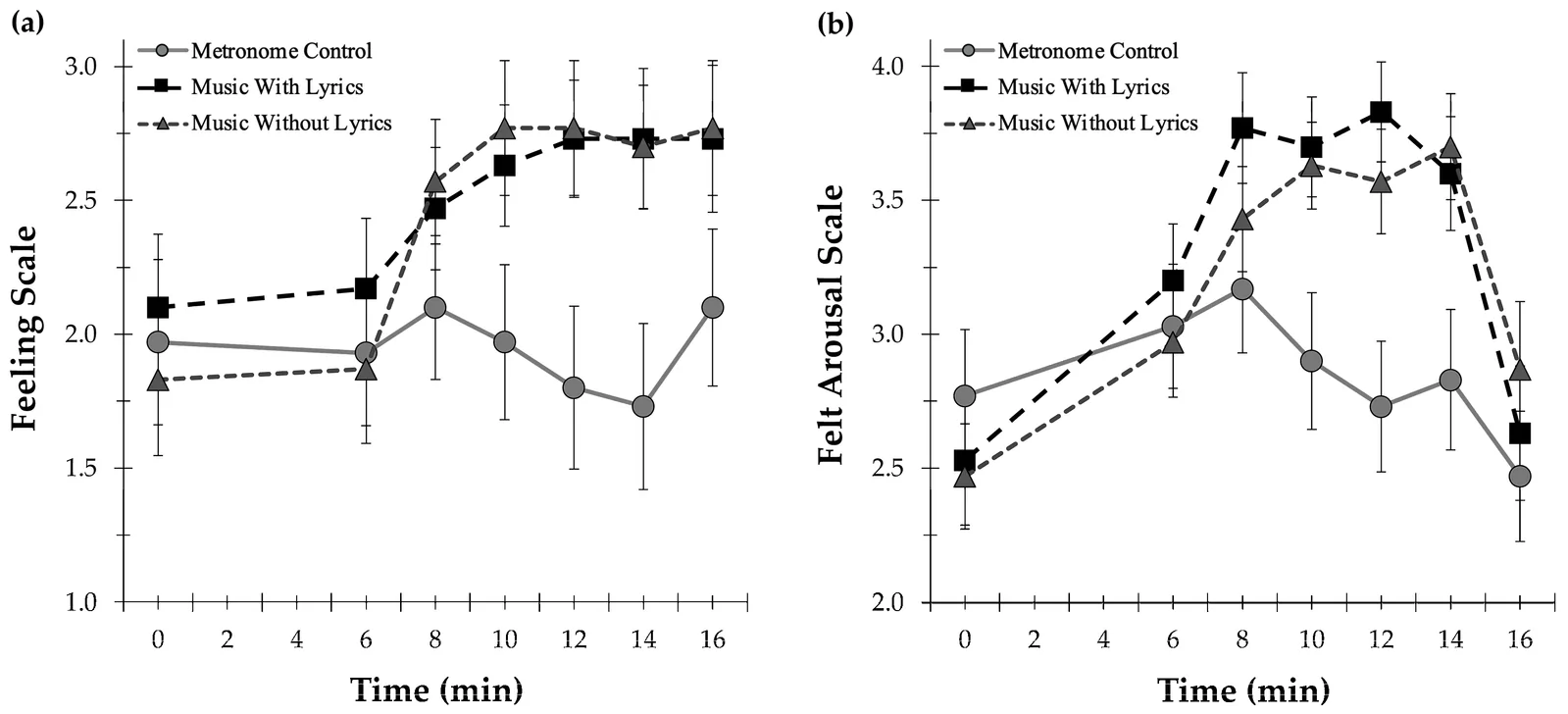
Average (**a**) affective valence (FS) and (**b**) arousal (FAS) responses across a 16-min cycling trial performed during music with lyrics, music without lyrics, and metronome control conditions. For clarity of interpretation, the y-axes are truncated and do not reflect anchor points.

The RM-ANOVA for the FS revealed Condition, *F*(2, 28) = 5.20, *p* = 0.012, *η*^2^*_p_* = 0.27, and Time, *F*(4, 26) = 7.30, *p* < 0.001, *η*^2^*_p_* = 0.53, main effects. Follow-up Bonferroni-corrected *t* tests indicated significantly higher affective valence during ML (*M* = 2.55, *SE* = 0.22) and MNL (*M* = 2.53, *SE* = 0.23) conditions relative to the MC (*M* = 1.91, *SE* = 0.28) condition. No differences were found between the two music conditions (*p* > 0.05). Collapsed by condition, the Time main effect showed affective valence was higher during the 8-min (*M* = 2.38, *SE* = 0.21), 10-min (*M* = 2.46, *SE* = 0.21), 12-min (*M* = 2.43, *SE* = 0.22), and 14-min (*M* = 2.39, *SE* = 0.22) time points relative to the 6-min time point (*M* = 1.99, *SE* = 0.24). The main effects were superseded by a Condition × Time interaction, *F*(8, 22) = 2.46, *p* = 0.045, *η*^2^*_p_* = 0.47. Decomposition of the interaction indicated steady increases in affective valence across time points in the ML and MNL conditions. In the MC condition, affective valence slightly increased between the 6- and 8-min time points followed by a consistent decrease across the 10-, 12-, and 14-min time points (Figure 4).

#### 3.1.5. Arousal (FAS)

Condition, *F*(2, 28) = 7.57, *p* = 0.002, *η*^2^*_p_* = 0.25, and Time, F(4, 26) = 6.56, *p* < 0.001, *η*^2^*_p_* = 0.50, main effects were found for the FAS. Follow-up tests indicated that the highest arousal responses were recorded in the ML (*M* = 3.62, *SE* = 0.18) and MNL (*M* = 3.46, *SE* = 0.17) conditions relative to the MC (*M* = 2.93, *SE* = 0.24) condition, with no significant difference between the two music conditions. For the Time main effect, arousal was lowest at the 6-min (*M* = 3.07, *SE* = 0.20) time point, increased at the 8-min (*M* = 3.46, *SE* = 0.19) time point, and began to decrease at the 10-min (*M* = 3.41, *SE* = 0.17), 12-min (*M* = 3.38, *SE* = 0.18), and 14-min (*M* = 3.38, *SE* = 0.20) time points. A significant Condition × Time interaction, *F*(8, 22) = 5.00, *p* = 0.001, *η*^2^*_p_* = 0.65, superseded these main effects. Decomposition of the interaction revealed that arousal was significantly higher in the ML and MNL conditions at the 10-, 12-, and 14-min time points relative to the MC condition. Additional comparisons indicated that arousal was significantly higher in the ML condition at the 8-min time point relative to the MC condition, with no differences observed between the ML and MNL conditions (Figure 4).

#### 3.1.6. Planned ML Versus MNL Contrasts

Paired contrasts of the ML and MNL conditions were computed for each outcome averaged across the 6–14 min analytic window. For motivation (MS), *M*_ML_ = 5.51 and *M*_MNL_ = 5.45; mean difference = 0.06, 95% CI [−0.33, 0.45], *t*(29) = 0.31, *p* = 0.757, *d_z_* = 0.06 (trivial). For affective valence (FS), *M*_ML_ = 2.55 and *M*_MNL_ = 2.53; mean difference = 0.01, 95% CI [−0.39, 0.42], *t*(29) = 0.07, *p* = 0.946, *d_z_* = 0.01 (trivial). For arousal (FAS), *M*_ML_ = 3.62 and *M*_MNL_ = 3.46; mean difference = 0.16, 95% CI [−0.09, 0.41], *t*(29) = 1.32, *p* = 0.198, *d_z_* = 0.24 (small). For perceived exertion (RPE), *M*_ML_ = 12.59 and *M*_MNL_ = 12.36; mean difference = 0.23, 95% CI [−0.13, 0.60], *t*(29) = 1.30, *p* = 0.203, *d_z_* = 0.24 (small). Across all four outcomes, ML versus MNL differences were non-significant, and standardized effects were trivial to small. These non-significant contrasts should not be interpreted as evidence of statistical equivalence as the 95% confidence intervals span both positive and negative values, and formal equivalence bounds were not pre-specified. The pattern is instead consistent with the interpretation that, under the specific stimuli and protocol used, adding lyrics to the music track produced at most a small incremental effect on the measured psychological outcomes.

## 4. Discussion

The primary aim of this study was to examine the effects of lyrics in music on motivation during moderate-intensity cycling. A secondary aim was to examine the effects of music on affective valence, arousal, and perceived exertion. Consistent with previous research (Elliott et al., 2004; Hutchinson et al., 2011; Karageorghis et al., 2013), participants reported significantly higher motivation, affective valence, and arousal during exercise in both music conditions relative to the metronome control (MC) condition, replicating the well-established beneficial effect of music during exercise (Karageorghis & Priest, 2012a, 2012b). The theoretically central comparison between music with lyrics (ML) and music without lyrics (MNL), by contrast, produced trivial-to-small, non-significant differences on all four outcomes. We interpret this pattern, within the context of the specific stimuli and protocol used in the present study, as a non-detectable psychological benefit of the lyric version relative to the instrumental version. Because the confidence intervals around the ML-MNL differences span both positive and negative values and no formal equivalence bounds were pre-specified, we do not interpret the null contrasts as evidence that ML and MNL are equivalent in general. We only report that no reliable ML-MNL difference was detected in this design.

Several features of the current design plausibly converged to attenuate any lyric-specific effect on the measured outcomes, and these should be considered together rather than in isolation. First, the selected track was highly familiar to participants (mean song familiarity 4.7/5.0). High familiarity may enable covert rehearsal of lyrics during the instrumental condition (Ali & Peynircioğlu, 2006; Juslin, 2009), which would attenuate the psychological distinction between ML and MNL by importing lyrical content into the instrumental condition mentally rather than acoustically. Second, the 4-min track was looped to fill the 8-min bout, and this repetition may have blunted the semantic impact of the lyrics during the second half of the bout by producing rapid stimulus habituation and reducing the novelty of the lyric content. Third, the metronome was overlaid across all three conditions to standardize cadence. While this was central to internal validity (permitting a clean lyric-content contrast), a repetitive artificial auditory cue may itself produce a ceiling or habituation effect on the psychological variables of interest, potentially masking subtle psychophysiological differences between the lyric and instrumental tracks. Fourth, the acoustic and rhythmic properties of music (e.g., tempo, timbre, harmonic content, positive associations with a familiar track) are largely preserved when lyrics are removed and are themselves known to elicit hedonic and motivational responses independent of semantic content (Bishop et al., 2007; Karageorghis, 2016; Salimpoor et al., 2015). Taken together, these features suggest that the null ML-MNL contrast should be interpreted as design-specific such that any incremental effect of lyrics may have been operating on top of a lyric-like signal already available to participants (via mental rehearsal), against a familiar and repeating acoustic background, and in the presence of a continuous metronome cue. Studies using unfamiliar tracks, single-play (i.e., non-looped) exposures, no metronome overlay, and post-condition assessments of lyric interpretation are needed before broader claims about the role of lyrics in exercise music can be tested.

Two mechanisms may plausibly account for these results, though the present study did not directly measure either of them. First, music is theorized to dissociate attention from interoceptive cues of effort (Rejeski, 1985; Hutchinson & Karageorghis, 2013). Second, the hedonic and emotional qualities of music are proposed to elevate affective valence and perceived activation independent of perceived exertion (Karageorghis & Priest, 2012a). Lyrics may provide additional semantic and emotional cues (e.g., empowering messages, familiar phrases) that support goal-directed cognition (Karageorghis, 2016). Because ML-MNL differences were not detected in the present sample, one plausible interpretation is that, for familiar popular tracks at moderate intensity, the acoustic and rhythmic properties of music may account for a larger share of the observed responses than lyrical content, with any incremental effect of lyrics being small under these conditions. This interpretation is offered as a hypothesis to be tested in future work rather than as a conclusion supported by direct measurement.

Related to the present findings, participants reported increased positive feelings of affect and higher levels of motivation during both music conditions (Marshall, 2017). Although some prior work (e.g., Potteiger et al., 2000; Terry et al., 2020) has reported reductions in RPE during music conditions, the present study did not detect condition-related differences in perceived exertion. Findings are consistent with previous studies assessing RPE across exercise conditions at a similar intensity. Tenenbaum et al. (2004) found similar RPE responses during music and no-music conditions while participants performed treadmill running despite reporting higher levels of motivation to continue the exercise bout. Similarly, Yamashita et al. (2006) found no significant differences in RPE during music and no-music conditions while performing an acute bout of cycling at 60% VO_2peak_. Together, these results suggest that listening to music may enhance psychological responses independent of RPE.

Similar to previous investigations (Boutcher & Trenske, 1990; Terry et al., 2020), increases in affective valence for both music conditions compared to the metronome control (MC) condition were found. This may be explained by several factors previously suggested in the music and exercise performance literature. The current study utilized a metronome-paced cadence of 65 RPM (half the song tempo of 130 BPM). Despite increases in resistance to achieve moderate-intensity exercise, entrainment (i.e., synchronization) to music rhythm may have led to more efficient movement patterns (Bacon et al., 2012; Terry et al., 2012), potentially optimizing arousal and leading to enhanced affective responses. However, cadence was standardized via a metronome across conditions, reducing the likelihood that entrainment alone explained the affective differences. Thus, affective improvements were likely driven by the motivational or hedonic qualities of the music rather than biomechanical changes. Specifically, music in both lyric and instrumental conditions likely elevated affective valence and perceived activation through affect-inducing acoustic features (e.g., tempo, timbre, harmonic content) and positive associations with familiar tracks (Bishop et al., 2007; Karageorghis, 2016; Salimpoor et al., 2015). These hedonic and motivational pathways are largely preserved when lyrics are removed, which may help explain the minimal differences observed between ML and MNL conditions.

Several limitations warrant consideration (Marshall, 2017). First, exercise intensity was standardized via heart rate, but ergometer resistance was manually adjusted to maintain the target range. Some session-to-session variability in mechanical workload cannot be excluded, and individual differences in training status may have introduced unmeasured variance in work output. Future studies should either standardize resistance and adjust intensity via speed alone or normalize intensity to ventilatory threshold via a preceding graded exercise test, which may be especially important for affective outcomes (Ekkekakis et al., 2004). A cycle ergometer with a digital workload readout would further improve precision. Whether and how music effects differ between trained and untrained participants under standardized versus unstandardized intensity also remains an open question (Karageorghis & Priest, 2012a).

Second, participants were instructed to pedal in time with a metronome set to 65 BPM (half the tempo of the selected track). Standardized cadence limited examination of performance outcomes and may reduce external validity. Although this approach strengthened internal control, it may not reflect typical exercise behavior. Future research should consider removing this constraint to enhance external validity. Furthermore, allowing self-selected tempo and cadence may yield different psychological responses. Along these lines, past research suggests that it may be more beneficial to include self-selected music versus prescribed music to increase positive responses (Razon et al., 2009; Terry et al., 2020). However, such music has generally not been selected with explicit reference to its lyrical content nor has it been selected while performing a similar exercise bout, whereas in the current study, the track was determined in Part 1 according to its motivational properties by participants of a similar socio-cultural background and age profile to participants who completed Part 2 (Karageorghis & Terry, 1997) and during a similar bout of exercise.

Finally, neither the meaning of the lyrics nor how the participants interpreted them was directly assessed. We acknowledge this as a major limitation because lyrical content is semantically and emotionally heterogeneous. Grouping tracks as “with” or “without” lyrics does not capture variation in lyrical meaning, valence, or personal resonance (Juslin, 2009; Ali & Peynircioğlu, 2006). Conclusions about lyrics as a category are therefore necessarily tentative, and future work should incorporate content analysis of lyrics and participant interpretation to meaningfully test their psychological effects. Past research examining music, emotion, and lyrics shows that the interpretation of the lyrics influences the overall emotional experience of music (Juslin, 2009). Assessing lyrical interpretation may clarify mechanisms linking lyrics and psychological responses. Furthermore, participants reported a high level of familiarity with the selected song. Coupled with its popularity, covert rehearsal of lyrics during the instrumental condition may have attenuated between-condition differences. For future studies, we suggest that researchers select fewer familiar songs with high motivational qualities or have participants select songs from a list that they view as having high motivational content.

## 5. Conclusions

In sum, both music conditions increased motivation, affective valence, and arousal during acute moderate-intensity aerobic exercise relative to the metronome control, replicating the established beneficial effect of music during exercise. Under the specific conditions tested (a single, highly familiar popular track, a metronome overlaid across all conditions, an 8-min moderate-intensity cycling bout, and a young university sample), no additional psychological benefit was detected for the lyric version of the track relative to its instrumental version. This null contrast is bounded by the design and should not be generalized as evidence that lyrics do not contribute to exercise-related psychological responses.

## Figures and Tables

**Table 1:** BMRI-2 scores and BPM (*M* ± *SD*) for music selection ^1^.

Track	BMRI-2	Vocals/Lyrics	BMRI-2 + Vocals/Lyrics	BPM
1	25.38 ± 8.88	8.82 ± 3.09	34.19 ± 11.95	125
2	33.31 ± 8.88	10.68 ± 3.20	44.08 ± 12.03	130
3	21.30 ± 8.32	6.93 ± 2.57	28.26 ± 10.74	128
4	27.69 ± 10.83	8.68 ± 3.28	36.42 ± 14.09	129
5	26.73 ± 11.41	8.46 ± 3.70	35.31 ± 15.15	126

^1^ BMRI-2 = Brunel Music Rating Inventory-2; BPM = beats per minute; Track 1 = *Wake Me Up* by Avicii; Track 2 = *Boom Boom Pow* by Black Eyed Peas; Track 3 = *Work Hard Play Hard* by Tiesto; Track 4 = *Instant Replay* by Dan Hartman; Track 5 = *Reflection* by Jacob Plant featuring Example.

**Table 2:** Participant characteristics (*M* ± *SD*) overall and by sex.

Measure	Male	Female	Total
*n*	11	19	30
Age (years)	21.7 ± 2.3	20.6 ± 3.3	21.03 ± 2.9
BMI (kg/m^2^)	24.7 ± 4.2	22.7 ± 3.9	23.4 ± 4.1
Height (cm) ^1^	180.6 ± 9.4	164.6 ± 7.6	170.4 ± 11.3
Weight (kg) ^1^	80.5 ± 14.4	61.5 ± 12.3	68.4 ± 15.9
Song Familiarity (1–5 scale) ^1^	4.4 ± 0.8	4.95 ± 0.2	4.7 ± 0.6
Song Enjoyment (1–5 scale)	3.3 ± 1.1	4.0 ± 1.0	3.7 ± 1.1

^1^ Significant difference, unpaired Student’s *t*-test between male and female participants, *p* < 0.05. BMI = body mass index; kg = kilogram; m = meter; cm = centimeter.

## Data Availability

The data presented in this study are available on request from the corresponding author. The data are not publicly available due to privacy and ethical reasons.

## Abbreviations

The following abbreviations are used in this manuscript:

ACSM	American College of Sports Medicine
ANOVA	Analysis of Variance
BMI	Body Mass Index
BMRI-2	Brunel Music Rating Inventory-2
BPM	Beats per Minute
cm	Centimeter
ES	Effect Size
FAS	Felt Arousal Scale
FS	Feeling Scale
HR	Heart Rate
HR_max_	Maximum Heart Rate
kg	Kilogram
M	Mean
m	Meter
MC	No Music Control
min	Minutes
ML	Music with Lyrics
MNL	Music without lyrics
MS	Motivation Scale
PAR-Q	Physical Activity Readiness Questionnaire
RM-ANOVA	Repeated Measures-Analysis of Variance
RPE	Rate of Perceived Exertion
RPM	Revolutions per Minute
s	Seconds
SE	Standard Error
SD	Standard Deviation
